# A Simple, Rapid, Fluorometric Assay for Dopamine by In Situ Reaction of Boronic Acids and *cis*-Diol

**DOI:** 10.1155/2019/6540397

**Published:** 2019-03-27

**Authors:** Xiaoxia Liu, Miaomiao Tian, Wenmei Gao, Jinzhong Zhao

**Affiliations:** ^1^College of Arts and Sciences, Shanxi Agricultural University, Jinzhong, Shanxi 030801, China; ^2^Key Laboratory of Nanobiosensing and Nanobioanalysis at Universities of Jilin Province, Department of Chemistry, Northeast Normal University, Changchun, Jilin 130024, China

## Abstract

An efficient, sensitive, and low-cost method has been developed for turn-on fluorescence sensing of dopamine (DA). The method relies on the rapid reaction of DA and 3-Hydroxyphenylboronic acid (3-HPBA) via specific recognition between boronic acids and *cis*-diol of DA in alkaline solution. The reaction product shows an excitation wavelength of 417 nm and the maximum emission peak at 470 nm. The proposed method allows the determination of DA in the range of 50 nM–25 *μ*M, and the whole detection can be completed within 5 minutes. Furthermore, the presented approach has good selectivity and has been successfully applied to DA sensing in human serum samples, showing great potential in clinical diagnosis.

## 1. Introduction

Dopamine (DA) is a significant catecholamine neurotransmitter which is used for message transfer between neurons and plays an important role in the nervous activity [1]. Abnormal DA concentrations in the brain may lead to several diseases such as parkinsonism, anorexia, and schizophrenia. Many DA-based drugs are widely used to treat these diseases [2]. The DA concentration ranges widely from 0.1 *μ*M to 1.0 mM in biological liquid [3]. Hence, a convenient and sensitive method for DA measurement is highly desirable for researching human physiological functions.

Until now, many techniques have been developed to detect DA, such as colorimetry [4–6], electrochemistry [7, 8], fluorescence spectroscopy [9–12], mass spectrometry [13], chemiluminescence [14], and high-performance liquid chromatography (HPLC) [15]. While significant progresses in detection of DA have been made using these methods, there are still some disadvantages and limitations. For example, the colorimetric method is limited by the low sensitivity or the low selectivity; the electrochemical method often suffers from the interferences of ascorbic acid and uric acid owing to the similar oxidation potential; HPLC and mass spectrometry are sensitive analytical methods, and they require expensive instrumentation and expertise for operation. Attractively, the fluorescence-based DA detection technique has attracted many attentions for its advantages including high sensitivity, facile operation, wide detection range, and high selectivity. The fluorescence method for DA detection is mainly based on fluorescence quenching (turn-off) or fluorescence enhancement (turn-on) strategy. The fluorescence quenching strategy is based on the oxidation of DA to produce quinine [16] or the photoinduced electron transfer (PET) process between the fluorescence materials and DA molecule [1, 17, 18]. The fluorescence enhancement strategy is more preferable than the quenching strategy, because it has higher selectivity due to the fluorescence is only enhanced or restored by interaction with preferred analyte. In addition, it is easy to observe the signal in a dark background rather than in a bright background [19].

Boronic acid as a Lewis acid can bind with 1,2-diols or 1,3-diols in aqueous solution reversibly to form five or six-membered cyclic ester [20–23]. As a result, the generation of the ester can change fluorescence significantly. Yoon and Czarnik firstly used boronic acid as a fluorescent chemosensor to detect sugar in 1992 [24]. Since then, the specific recognition abilities of boronic acids against 1,2-diols or 1,3-diols including saccharides, catecholamines, and glycosylated biomolecules have been extensively investigated. Until now, a large number of fluorescent probe with boronic acid have been developed to detect DA. For example, Qin's group [25] used boronic acid functionalized boron dipyrromethene (BABDP) as a fluorescent probe for the detection of DA. The fluorescence of BABDP can be strongly quenched by DA due to the PET. Detection of DA was ranged from 10^−8^ M to 10^−2^ M. Zhou et al. [26] developed a novel fluorescence sensor for DA determination based on molecularly imprinted graphene quantum dots and poly(indolylboronic acid) composite (MIPs@ PIn-BAc/GQDs). The covalent binding between the catechol group of DA and boronic acid leads to aggregation and fluorescence quenching of the MIPs@ PIn-BAc/GQDs. Chibac et al. [27] synthesized two copolymers containing different boronic acid derivatives. Both copolymers were tested as fluorescent sensors for detection of diols at physiological pH and to investigate the role of the boronic acids in the fluorescence response mechanism. The results showed that the addition of DA leads to a fluorescence quenching of the copolymer solutions, and the hydroxyl groups in 1, 2 positions played a determinant role in the quenching mechanism. All the previous studies can be divided into two approaches, i.e., modification of boronic acid molecules on the surface of fluorescent particles and design of fluorescent molecules containing boronic acid. Both approaches are turn-off strategy, and the preparation is time-consuming, uneconomical, and sometimes environmentally unfriendly.

**Figure 1:**
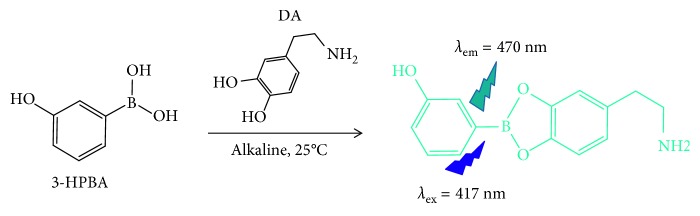
DA reacting with 3-HPBA to synthesize five-membered cyclic ester.

In this paper, we developed a sensitive and efficient method for DA detection by in situ method. The method relies on the reaction between 3-Hydroxyphenylboronic acid (3-HPBA) and DA in alkali condition to generate fluorescence (Figure 1). For detection, the reaction product is excited at the wavelength of 417 nm, leading to the maximum emission peak at 470 nm. A good linear relationship was obtained between the fluorescence intensity and the DA concentration. Furthermore, 3-HPBA has high selectivity for DA detection, and the whole detection can be completed within only 5 minutes without any pretreatments. The proposed method has been successfully applied for the reliable detection of DA in human serum samples.

## 2. Materials and Methods

### 2.1. Reagents and Chemicals

DA hydrochloride and 3-HPBA were purchased from Sigma-Aldrich (U. S). Tyrosine, ascorbic acid, alanine, serine, glycine, tryptophan, lysine, cysteine, glucose, glutathione, norepinephrine, saccharide, and sodium hydroxide were obtained from Aladdin Co., Ltd. (Shanghai, China). All reagents were of analytical grade and used without further purification.

### 2.2. Measurement

The UV absorption spectra were recorded on a UV-2450 spectrophotometer (Shimadzu). The fluorescence emission spectra were performed on an F-7000 spectrophotometer (Hitachi, Japan), and the slit widths of excitation and emission were set at 10.0 nm with a 700 V PMT voltage.

### 2.3. Detection of DA

Different concentration (0 to 400 *μ*M) of DA aqueous solution (0.5 mL) was mixed to 0.1 mL 8 mM 3-HPBA solution. Then, Na_2_CO_3_ buffer (0.4 mL 25 mM, pH = 10.5) was introduced into a 2 mL Eppendorf tube. After incubated for 5 min at 25°C, the fluorescence spectra of the reaction solution were recorded.

### 2.4. Interferences Study

Some coexistence substances will affect the detection of DA. To evaluate the feasibility and selectivity of the proposed method, DA and the interference of possible chemicals were mixed to obtain a set of solutions, each one with 20 *μ*M DA and 1 mM interference.

### 2.5. Real Sample Analysis

To evaluate the capability of the proposed method in real sample analysis, we analyzed the DA concentrations in human serum samples. The healthy adult human serum samples were collected in a heparin anticoagulated tube and treated by centrifugal ultrafiltration at 3000 rpm for 15 min at room temperature to remove haemocytes. Then, 0.20 mL of different concentrations of DA was added into the serum samples (0.20 mL) to prepare the spiked samples. These samples were detected by the aforementioned method.

## 3. Results and Discussion

### 3.1. Fluorescent Properties

**Figure 2:**
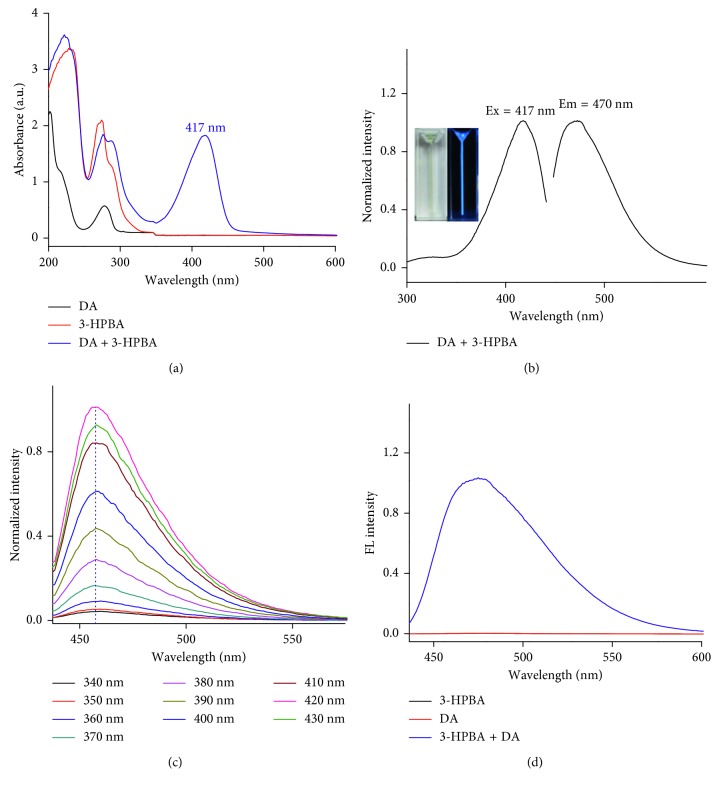
(a) UV-Vis absorption spectrums of DA, 3-HPBA, and product. (b) The excitation and emission spectrums of the product. (c) Fluorescence emission spectrum was excited at different wavelengths from 340 nm to 430 nm. (d) Fluorescence emission spectrum of DA, 3-HPBA, and product.

To investigate the properties of the fluorescent product, we first studied the UV-Vis absorption spectra of DA, 3-HPBA, and product. From Figure 2(a), we can see that both 3-HPBA and DA have an apparent absorption peak around 280 nm. After a period of reaction, the characteristic absorption peak of DA and 3-HPBA is remained, and a new strong peak appears at 417 nm, demonstrating that a new five-membered cyclic ester has been generated. As shown in Figure 2(b), the product exhibited the maximal emission at 470 nm when excited at 417 nm. The insert photographs are the fluorescence of product solution under natural light (left) and UV (right) lamp, respectively. In Figure 2(c), we show fluorescence signal as a function of excitation wavelength. There is no shift of the fluorescence peak while changing the excitation from 340 nm to 430 nm, indicating that the new product has a unique fluorescence property and monochromaticity. From Figure 2(d), we can see that both 3-HPBA and DA generate no fluorescence, indicating that neither the excess of 3-HPBA nor DA has effect on the fluorescence detection.

**Figure 3:**
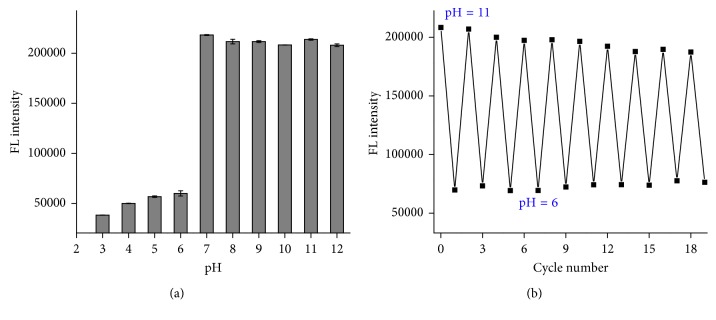
(a) Fluorescence intensity as a function of pH from 3–12 and (b) the reversibility of the reaction between the boronic acid and DA in pH 6 and 11. The pH of buffer solution was adjusted by HCl or Na_2_CO_3_.

We then studied the pH effect on the fluorescence intensity of the new product. The fluorescence intensities of the new product were monitored in buffer solutions with different pH values (3–12). As one can see from Figure 3(a), the fluorescence intensity of complex is stable in the pH range of 7–12 and dramatically decreases as the pH is decreased to lower than 6. The possible reason is that the formed five-membered cyclic ester between the boronic acid and *cis*-diols of DA is a reversible covalent complex in an alkaline/acidic aqueous solution. In order to investigate the reversibility of the reaction, we changed the pH value periodically from 11 to 6 and measured the fluorescence intensity at each pH value.  Figure 3(b) shows the cycles can be repeated ten times without fatigue. Our results prove that the binding of boronic acid and *cis*-diols is reversible, and the fluorescence intensity for DA detection should be performed in a basic pH condition.

### 3.2. Detection of DA

**Figure 4:**
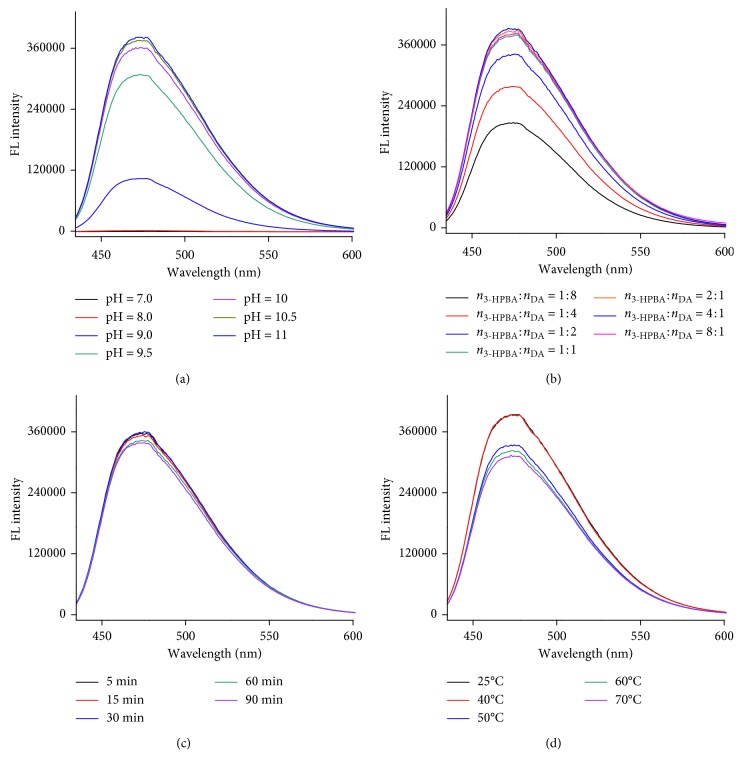
The effect of experimental conditions on the fluorescence intensity: (a) pH, (b) the ratio of 3-HPBA to DA, (c) reaction time, and (d) temperature. Other parameters were fixed, and 0.8 mM 3-HPBA and 100 *μ*M DA reacted in 10 mM Na_2_CO_3_.

Before performing the assay, we optimized several experimental conditions that may affect fluorescence intensity, including pH, the amount of 3-HPBA, reaction time, and temperature. As shown in Figure 4(a), in a neutral or weak alkaline solution, the reaction solution did not generate any fluorescence. The fluorescence intensity of the complex increases with the pH value increasing from 9.0 to 11. When pH is equal to or higher than 10.5, the fluorescence intensity reached its maximum value. So, a pH of 10.5 was chosen as the optimal value for subsequent experiments. In order to evaluate the effect of the amount of 3-HPBA on the resulting fluorescence intensity, the ratio of 3-HPBA to DA (*n*/*n*) was varied from 1 : 8 to 8 : 1, and other parameters were fixed. The results are shown in Figure 4(b). As the ratio of 3-HPBA to DA (*n*/*n*) increased, the fluorescence intensities increased in the range from 1 : 8 to 1 : 1, subsequently, maintained almost constant when increased the amount of 3-HPBA. Thus, the final concentration of 3-HPBA was higher than DA in the subsequent experiments.  Figure 4(c) shows that the reaction is fast, which only needs 5 min to reach the equilibrium. In Figure 4(d), we show the effect of reaction temperature on the fluorescence intensity. As the temperature is increased, the fluorescence intensity decreases. So, we performed the reaction at room temperature.

**Figure 5:**
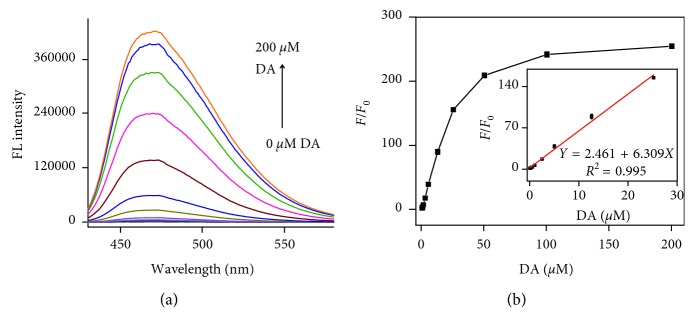
(a) Fluorescence spectrum at different DA concentrations (0–200 *μ*M) and (b) dependence of the FL intensity at 470 nm on DA concentration.

In optimal conditions, we used the in situ reaction between 3-HPBA and DA to produce strong fluorescence intensity to detect the DA. The fluorescence intensity at 470 nm as a function of DA concentration is shown in Figure 5. There is a good linear relationship between the fluorescence intensity and DA concentration from 50 nM to 25 *μ*M (Figure 5(b)). The limit of detection (LOD) is calculated to be 20 nM using the calibrate line.

### 3.3. Study of Interferences

To investigate the selectivity of the proposed method, we studied the effect of various possible interfering chemicals including amino acids (Cys, Lys, Trp, Ala, and Ser), glucose (Glu), ascorbic acid (Vc), glutathione (GSH), saccharides (Sar), and norepinephrine (NE). Various agents with a far higher than the physiological concentration were added to DA for interference testing.

**Figure 6:**
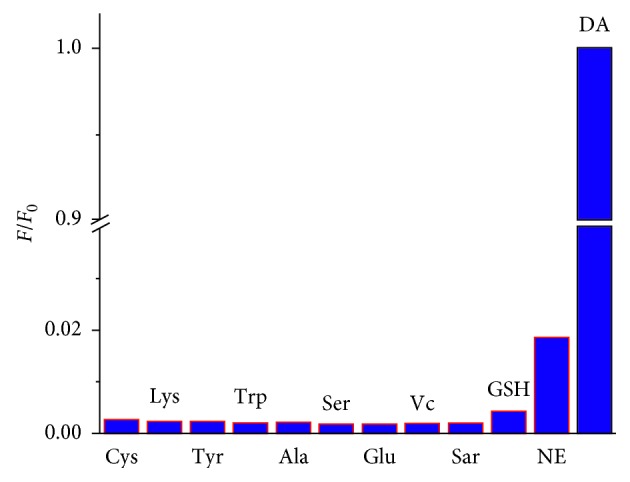
Effect of interferences on DA detection. The concentration of DA and other interferences is 20 *μ*M and 1 mM, respectively.

Figure 6 shows that all the interferences tested almost produce no fluorescent signal even at such a high concentration, indicating that this method for detecting DA has high selectivity. It is noteworthy that NE exhibits higher interference on the measurement of DA than other substances, which could be attributed to its similar structure as DA that can react with 3-HPBA. On the other hand, the maximal absorption of the NE product is at 488 nm instead of 417 nm (Figure S1). Thus, the interference of NE is small for the proposed method. As shown in Figure 6, the fluorescence intensity of NE is less than 2% as that of DA, indicating the acceptable selectivity of our method for DA determination.

### 3.4. Real Sample Analysis

To demonstrate the potential utility of the proposed method, we used two human serum samples to investigate the performance for DA detection. After these samples were treated with the procedures as in the experimental section, a known concentration of DA was added to study the recovery. The results are shown in Table 1. Satisfactory recoveries from 85.4% to 108.0% were obtained in the spiked samples, and the relative standard deviation (RSD) is less than 3.81% (*n*=3). This indicates that this method has acceptable accuracy and reproducibility in detecting DA in real samples.

## 4. Conclusions

In summary, we have developed a sensitive and efficient turn-on fluorescence method for DA detection. This method is based on the in situ reaction between 3-HPBA and DA. Linear dependence of the fluorescence intensity on the DA concentration is obtained from 50 nM to 25 *μ*M. The whole detection can be completed within only 5 minutes without any pretreatments. In addition, the method exhibits high specificity among other analogues and is successfully applied in real sample detection with good accuracy. Our study indicates that the approach is very promising in clinical applications.

## Figures and Tables

**Table 1:** Determination of DA in human serum samples.

Sample	Spiked (*μ*M)	Measured (*μ*M)	Recovery (%)	RSD (%), *n*=3
Serum-1	1	1.03	103.0	2.05
	5	4.27	85.4	3.17
	10	9.51	95.1	3.81

Serum-2	1	1.08	108.0	1.57
	5	5.14	102.8	0.35
	10	9.45	94.5	1.69

## Data Availability

The data used to support the findings of this study are included within the article, and any further information is available from the corresponding author upon request.

## Supplementary Materials

Supplementary Materials Figure S1. The excitation and emission spectra of 3-HPBA/NE complex.
