# Supplementary figures and images for: A Simple, Rapid, Fluorometric Assay for Dopamine by In Situ Reaction of Boronic Acids and cis-Diol
Source: J Anal Methods Chem. 2019 Mar 27;2019:6540397. doi: 10.1155/2019/6540397 (PMC6458919; doi:10.1155/2019/6540397)

## Slide 1
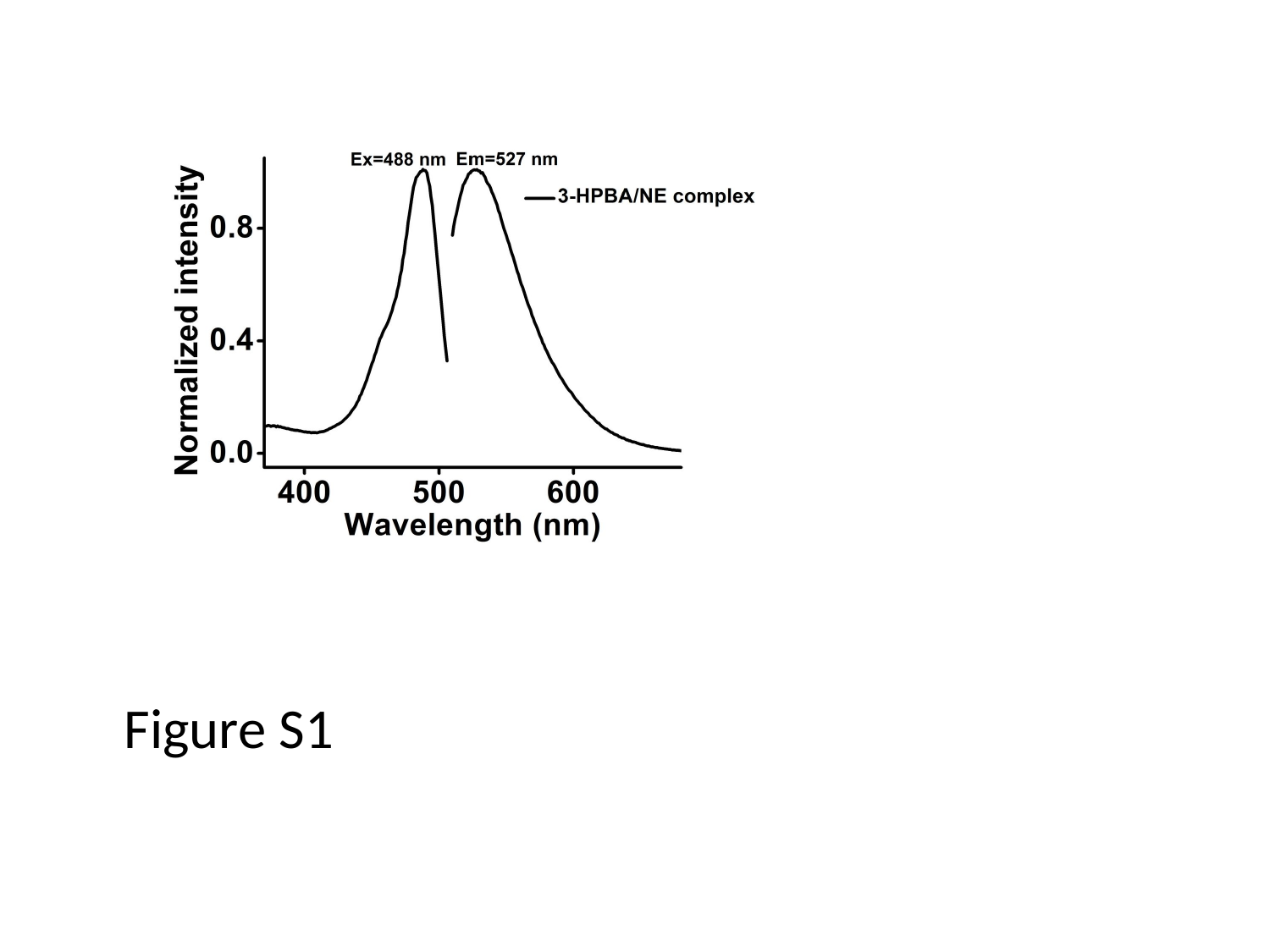

Figure S1

Supplement: Supplementary Materials — Figure S1. The excitation and emission spectra of 3-HPBA/NE complex. [file 6540397.f1.pptx]
